# Exploring the Potential of Agro-Nanotechnology in African Agriculture: A Path to Sustainable Development—Systematic Review

**DOI:** 10.1155/tswj/9073364

**Published:** 2025-03-17

**Authors:** Yohannes Gelaye

**Affiliations:** Department of Horticulture, College of Agriculture and Natural Resources, Debre Markos University, Debre Markos, Amhara, Ethiopia

**Keywords:** agro-nanotechnology, nanofertilizers, nanopesticides, nanosensors, precision agriculture

## Abstract

Agro-nanotechnology—the application of nanotechnology in agriculture—holds immense promise for addressing main challenges in African agriculture and promoting sustainable development. This review provides a comprehensive analysis of how agro-nanotechnology is being utilized across Africa, emphasizing its potential to revolutionize various aspects of agricultural practices on the continent. Firstly, the utilization of nanomaterials such as nanoparticles, nanofertilizers, and nanopesticides offers opportunities for enhancing nutrient management, improving soil health, and increasing crop productivity in diverse agroecosystems across Africa. Nanofertilizers, with their controlled release mechanisms, facilitate efficient nutrient uptake by plants, thereby reducing nutrient losses and enhancing fertilizer use efficiency, which is crucial for resource-constrained smallholder farmers. Nanopesticides suggest improved efficacy in pest and disease control, reducing environmental harm compared to traditional pesticides. Their targeted delivery also minimizes off-target effects, which is crucial for Africa's food security. Nanosensors also enable real-time monitoring of soil and crop health, enhancing precision agriculture. Nanotechnology in postharvest management reduces food losses and improves safety. However, its adoption requires careful consideration of socioeconomic and regulatory factors to ensure equitable access and environmental safety. Collaborative efforts involving policymakers, researchers, farmers, and other stakeholders are crucial for harnessing the benefits of agro-nanotechnology while addressing potential risks and concerns. In conclusion, the integration of agro-nanotechnology into African agriculture presents a transformative opportunity to enhance productivity, resilience, and sustainability, contributing to the continent's efforts toward achieving food security, economic development, and environmental conservation.

## 1. Introduction

Agriculture is vital to the food and feed industries but faces challenges like climate change, resource depletion, poor infrastructure, and limited technology [1]. Challenges in agriculture hinder global productivity and international rankings, making it crucial to address these issues to combat poverty and hunger. Enhanced practices and technologies like nanotechnology are crucial for addressing rural poverty and nutritional deficiencies and promoting sustainability, with agro-nanotechnology being a key application [2]. Agricultural nanotechnology focuses on using nanomaterials and devices to boost productivity, optimize resources, and reduce environmental impacts in crop cultivation [3]. Agro-nanotechnology integrates diverse applications, including nanosensors for soil and crop monitoring, nanomaterials for controlling nutrient and pesticide release, and nanobiotechnology for enhancing plant growth and stress resilience [4]. It offers immense potential for revolutionizing agriculture in Africa [5]. Agro-nanotechnology utilizes nanomaterials and principles to enhance crop yields, improve nutrient delivery, enable precision farming, and mitigate environmental impacts [4]. This technology optimizes fertilizer and pesticide efficiency, reducing costs and environmental harm for smallholder farmers. Countries like Nigeria, Kenya, South Africa, and Egypt, with thriving agricultural sectors, tend to host active agro-nanotechnology research and development entities [6]. South Africa, Kenya, Nigeria, and Egypt spearhead agro-nanotechnology research with institutions like ARC, KALRO, NASENI, and universities advancing crop protection, soil management, and precision agriculture [6]. International collaborations with institutions worldwide could enhance agro-nanotechnology adoption in Africa, with nanosensors optimizing irrigation by monitoring soil moisture levels [7]. Targeted delivery of pesticides and fungicides reduces chemical usage, while nanoparticles help remediate polluted soils and enhance nutrient uptake for plants, particularly in nutrient-deficient soils [8]. Nanotechnology in African agriculture offers potential benefits, but thorough studies are essential to understand its long-term impact on soil, water, and ecosystems for sustainability [9, 10]. Developing regulatory frameworks for nanotechnology in African agriculture is crucial for ensuring safety, assessing efficacy, evaluating cost-effectiveness compared to traditional methods, and facilitating smallholder adoption through accessibility, affordability, and cultural acceptance [11]. Public awareness, stakeholder engagement, capacity building, knowledge transfer, and investment in education and technology programs collectively support successful adoption and uptake [12]. Blending agro-nanotech with traditional farming and indigenous knowledge boosts African farming sustainability and resilience [13]. Integrating modern nanotechnology with traditional practices can create more holistic agricultural development approaches, while bridging gaps and challenges requires interdisciplinary collaboration, research investment, and stakeholder engagement across Africa's agricultural value chain [14]. This approach can unlock the potential of agro-nanotechnology for food security, sustainable agriculture, and socioeconomic development. Thus, this review offers a new insight of how agro-nanotechnology is utilized in Africa, emphasizing its capacity to transform different facets of agricultural methods across the continent.

## 2. Methodology

To explore the potential of agro-nanotechnology in enhancing African agriculture and promoting sustainable development, a systematic review was conducted using multiple search engines, including ScienceDirect, Scopus, and Web of Science. Specific keywords such as “agro-nanotechnology,” “nanotechnology in agriculture,” “sustainable agriculture Africa,” and “nanofertilizers” were utilized to gather relevant studies. The search was restricted to peer-reviewed articles mainly published from 2002 to the present (2024), focusing on empirical data and analyses related to agro-nanotechnology's impact on African agriculture. Inclusion criteria were also employed to select studies that are directly relevant, methodologically rigorous, and impactful, while excluding articles that were not in English, unrelated to the topic, or duplicates. Data were extracted based on the study objectives, methods, and findings and synthesized by categorizing them such as nanofertilizers and nanopesticides. Also, publication trends and citation metrics were analyzed to understand the research landscape. The majority of the works concluded that agro-nanotechnology can significantly improve African agriculture by boosting crop yields, enhancing soil fertility, and optimizing nutrient utilization. Furthermore, its role in precision farming can control the delivery of fertilizers and pesticides, also strengthening crops against environmental stresses. Likewise, adopting nanotechnology-based solutions could support food security, promote sustainable farming practices, and aid in adapting to climate change, positioning it as a valuable approach for the future of agriculture in Africa.

## 3. Overview of Agro-Nanotechnology in Africa

**Figure 1:**
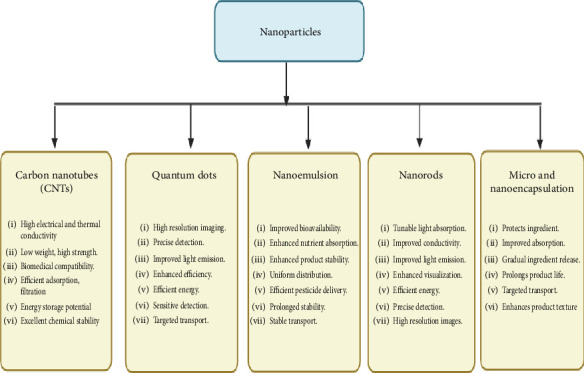
Nanoparticles and their functions.

Agro-nanotechnology in Africa addresses soil fertility, water scarcity, pest management, and crop productivity [15]. Nanofertilizers improve soil fertility by directly delivering nutrients to plants, reducing waste, and enhancing uptake efficiency, particularly in nutrient-deficient soils [16]. Smart irrigation and targeted pest control using nanotechnology tackle water scarcity and promote sustainability [17]. Nanomaterials, such as nanoparticles and nanocomposites, strengthen crop protection by providing physical barriers against pests and pathogens and releasing bioactive compounds to reduce crop losses [18, 19]. Precision agriculture facilitated by nanoscale sensors and imaging techniques monitors soil moisture, nutrient levels, and plant health in real time, enabling data-driven decisions for optimizing crop production while minimizing inputs and environmental impact [20]. Agro-nanotechnology holds promise for sustainable agriculture in Africa, mitigating agrochemical use and pollution, conserving water, and enhancing soil health [21]. Collaboration among researchers, policymakers, industry, and farmers is essential to maximize benefits, foster sustainable development, and overcome persisting challenges [22]. Policymakers and stakeholders are essential for advancing agro-nanotechnology through supportive regulations, funding, and infrastructure, despite facing challenges such as regulatory hurdles, high costs, limited awareness, and environmental concerns, while nanoparticles offer diverse applications including drug delivery, catalysis, imaging, sensing, and material reinforcement due to their unique properties (Figure 1).

## 4. Major Types of Agro-Nanotechnology in Africa

Agro-nanotechnology is a rapidly advancing field that applies nanotechnology to agriculture. The table outlines some of its key types (Table 1).

## 5. Graphics of Agro-Nanotechnology in Africa

Agro-nanotechnology could transform African agriculture, boosting crop yield, soil quality, and environmental sustainability [30]. Nanotechnology enhances African agriculture by improving nutrient delivery and pest control with nanofertilizers and nanopesticides, leading to increased crop yields and reduced chemical use [31]. Nanosensors enable precise monitoring of soil and environmental conditions, optimizing resource use and minimizing waste [32]. Additionally, nanotechnology aids in soil and water remediation, supporting sustainability and boosting food security in the region [33]. See the key graphics showcasing its potentials in Table 2.

## 6. Role of Agro-Nanotechnology for Climate Change Mitigation in Africa

Agro-nanotechnology, the application of nanotechnology in agriculture, holds promise for climate change mitigation in several ways [44]. Nanotechnology has advanced African agriculture by boosting crop yields through enhanced nutrient delivery and pest control with nanofertilizers and nanopesticides [45]. It also improves resource efficiency and reduces waste with precise monitoring via nanosensors [46]. Additionally, nanotechnology supports soil and water remediation, contributing to sustainable practices and increased food security [47]. Nanotechnology boosts crop yields by enabling controlled fertilizer release, targeted nutrient delivery, and ecofriendly nanoscale pesticides [48]. Nanostructured materials enhance water use in agriculture by improving soil moisture retention, reducing runoff, and enabling precision irrigation [49]. Nanomaterials can be used to enhance soil carbon sequestration, aiding in the removal of atmospheric carbon dioxide and mitigating climate change [50]. Nanotechnology advances materials for solar cells, energy storage, and biofuel catalysts, aiding renewable energy adoption and reducing greenhouse gas emissions [51]. Nanosensors monitor soil moisture, nutrient levels, and crop health, enabling precision agriculture that optimizes resources and reduces environmental impact [4]. Nanotechnology solutions enhance agricultural waste management by remediating contaminated soils and converting waste into biofuels or bioplastics [52, 53].

## 7. Agro-Nanotechnology Contribution to Postharvest Management in Africa

Agro-nanotechnology can transform postharvest management in Africa by extending shelf life with advanced packaging, real-time monitoring with nanosensors, and improving nutrient uptake and pesticide delivery [54]. It also offers solutions for safer pest management, stability through nanoencapsulation of bioactive compounds, and precise quality assessment with nanobiosensors [55]. Nanomaterials improve storage insulation in areas with unreliable power, but their responsible deployment is crucial for environmental impact, safety, and affordability for smallholder farmers. Agro-nanotechnology also enhances postharvest management in Africa by extending crop shelf life with advanced packaging and real-time monitoring using nanosensors [56, 57]. Nanocoatings protect produce and reduce spoilage, minimizing waste and the need for preservatives [58]. This technology improves food safety and quality and supports food security by reducing economic losses for farmers.

## 8. Agro-Nanotechnology and Other Scientific Disciplines in Africa

Agro-nanotechnology in Africa merges nanotechnology with agriculture, driving progress in farming, food security, and sustainability [59]. It leverages nanoscale manipulation to enhance crop production, pest control, and soil health [60]. Integrating nanotechnology with traditional agriculture leads to innovations like nanomaterial-based fertilizers and pesticides that boost crop yield and resilience, while also enhancing soil quality, reducing water use, and curbing pollution [3, 61]. Biotechnology intersects with agro-nanotechnology through enhanced genetic engineering, enabling targeted crop improvements [44]. Material science, engineering, and chemistry are pivotal, crafting tailored nanomaterial solutions for agricultural challenges [62]. Moreover, combining agro-nanotechnology with digital transformation can amplify their impact on African agriculture [63]. Nanosensors in soil transmit data wirelessly to mobile-accessible digital platforms, enabling remote field management and facilitating the dissemination of agro-nanotechnology solutions and farmer training [64, 65]. Leveraging these technologies can help African farmers overcome challenges and build a resilient, sustainable agricultural sector, making interdisciplinary collaboration essential for practical, accessible solutions.

## 9. Agro-Nanotechnology and Politics in Africa

The utilization of nanotechnology in agriculture, known as agro-nanotechnology, carries substantial promise for tackling food security issues in Africa [66]. Adoption depends on political factors, as regulations on nanotechnology can either facilitate or hinder its agricultural use [67]. Establishing clear regulatory frameworks is vital to ensure safe and responsible use while encouraging innovation and investment [68]. Political commitment is crucial for agro-nanotechnology, requiring government funding for research, infrastructure, and capacity building [69, 70]. African nations' reliance on international cooperation for accessing nanotechnology makes political relationships crucial for technology transfer and knowledge sharing [71]. Political decisions also affect socioeconomic factors influencing farmers' adoption of agro-nanotechnology, such as resource access and education [69]. Promoting inclusive development and addressing technology disparities can boost uptake, as political discourse on ethics and environmental impacts shapes public perception and regulatory actions [72, 73]. Political leaders play a pivotal role in fostering informed debates and decision-making processes [72]. Effective governance and leadership are crucial for realizing agro-nanotechnology's potential in enhancing food security and ensuring its safe, sustainable implementation in Africa [74]. The involvement of policymakers, researchers, and other stakeholders is crucial for shaping the research and development landscape in African agriculture [75]. Policymakers influence the direction and funding of agricultural R&D, while researchers contribute to innovation and the development of practical solutions [76]. Collaboration among these groups, along with engagement with farmers and industry experts, ensures that research addresses real-world challenges and supports effective implementation [77]. Thus, by working together, stakeholders can drive progress in agro-nanotechnology, ensuring that advancements benefit all levels of the agricultural sector and contribute to sustainable development across the continent.

## 10. Fates of Agro-Nanotechnology in Africa

### 10.1. Socioeconomic Accessibility

Agro-nanotechnology can address agricultural challenges but raises socioeconomic concerns, making access for small-scale farmers in developing nations crucial to avoid widening the gap with larger farms [78]. Affordability is a concern due to the high costs of nanotechnology, potentially leading to dependency and financial strain [44]. Environmental impacts and regulatory challenges, including safety and intellectual property rights, must be carefully managed [79]. Collaborative efforts to address societal concerns about health, ethics, and corporate control are crucial for equitable and sustainable agro-nanotechnology development.

### 10.2. Societal Acceptance and Ethical Considerations

Nanotechnology in agriculture offers potential benefits like increased crop yields, reduced pesticide use, and improved soil fertility [80]. Concerns about agro-nanotechnology include environmental impacts like nanoparticle accumulation and toxicity, as well as exacerbating social inequality due to limited access for small-scale farmers [44, 81]. Ensuring equitable access to nanotech solutions is crucial [82]. Additionally, there are health risks for workers and consumers exposed to nanomaterials [83]. Ethical implementation demands rigorous risk assessment, safety measures, and effective regulatory frameworks, including testing, labeling, and monitoring [42]. Respecting local values and traditions in Africa through meaningful stakeholder engagement is essential for ensuring long-term sustainability and ethics and addressing unintended consequences [84]. While agro-nanotechnology holds promise for African agriculture, ethical implications must be carefully managed to prioritize human and environmental well-being.

### 10.3. Regulatory Hurdles

Agro-nanotechnology promises benefits for African agriculture but faces regulatory hurdles due to insufficient guidelines and a lack of specific regulations, hindering risk assessment [10]. Regulatory bodies often lack expertise and infrastructure for thorough evaluations, requiring investment in training and technology [85]. Costly compliance and public awareness gaps pose safety concerns for small farmers, while trust building and stakeholder dialogue are crucial; harmonizing regulations with international standards is vital but complicated by differing priorities among African countries [86]. Multistakeholder collaboration is needed to navigate these challenges and realize the benefits of agro-nanotechnology in African agriculture.

### 10.4. Environmental Challenges

Agro-nanotechnology in Africa offers promising solutions for agricultural challenges but raises environmental concerns, as improper management of nanomaterials like nanoparticles and nanofertilizers could lead to soil and water contamination [17, 87]. These particles may harm soil microbes, plants, and aquatic life, potentially disrupting ecosystems [88]. Accumulation in organisms can lead to biomagnification, posing risks to higher trophic levels, including humans [89]. Monoculture farming, intensified by agro-nanotechnology, threatens biodiversity and increases vulnerability to pests [63]. Waste disposal of nanomaterials presents challenges, potentially exacerbating contamination [90]. Comprehensive risk assessments, monitoring, regulation, research for safer alternatives, and sustainable practices are essential for mitigating long-term environmental impacts.

### 10.5. Resistance Development

Limited research on resistance development from nanotechnology in agriculture suggests that nanopesticides and nanofertilizers could introduce selective pressures on pests and pathogens. Long-term monitoring and rigorous risk assessments are crucial for detecting potential resistance mechanisms and unintended consequences from the persistent use of nanomaterials in agriculture.

### 10.6. Risk of Nanoparticle Accumulation

The risk of nanoparticle accumulation in agro-nanotechnology, used to enhance crop yield and pest control, requires careful consideration, including in Africa [2]. Concerns focus on the environmental accumulation and unintended consequences of nanoparticles due to their persistence and ability to accumulate in soil, water, and plants [10]. This accumulation poses risks to ecosystems and human health, particularly in Africa, where agriculture is vital and environmental resources are fragile [91]. Mitigating these risks involves thorough nanoparticle impact assessments, responsible use, and investing in tailored research and regulation for sustainable agriculture in Africa [92].

## 11. Review Gaps and Future Perspectives

Early-stage research on agro-nanotechnology in African agriculture reveals gaps that need addressing for sustainable development, with environmental impact assessments crucial for evaluating nanoparticles' long-term effects on soil health, water quality, and ecosystem balance. Research should assess human health risks from ingestion, inhalation, and dermal contact and conduct long-term studies on toxicity and bioaccumulation in food crops and livestock, while understanding how nanoparticles affect nutrient uptake and stress tolerance in plants. Developing crop-specific nanotechnology solutions for African challenges, such as nanoparticle-based fertilizers and pesticides, requires addressing socioeconomic implications, adoption barriers, and regulatory frameworks for safe use. Building local research and innovation capacity is crucial for sustainable development and advancing agricultural practices in Africa.

## 12. Conclusion

In conclusion, the exploration of agro-nanotechnology presents a promising avenue for advancing African agriculture towards sustainable development. Agro-nanotechnology provides innovative solutions to key agricultural challenges in Africa, such as soil degradation, water scarcity, pest and disease management, and low crop productivity. By leveraging nanotechnology, African farmers can boost crop yields, conserve resources, minimize environmental impact, and strengthen food security for the continent's growing population. However, it is essential to proceed with caution, ensuring that the adoption of agro-nanotechnology is accompanied by thorough research, responsible implementation, and consideration of potential risks and ethical implications. Effective collaboration among scientists, policymakers, farmers, and other stakeholders is essential to fully harness the benefits of agro-nanotechnology, address concerns, and ensure equitable access for all farmers. Embracing agro-nanotechnology can lead Africa towards a more sustainable and prosperous agricultural future.

## Figures and Tables

**Table 1:** Types of agro-nanotechnology in Africa.

**Different categories of agricultural nanotechnology**	**Description of the role and contributions of the agro-nanotechnology in Africa**	**Ref.**
Nanofertilizers	Nanofertilizers boost plant nutrient absorption, enrich soil, and curb nutrient loss, thereby enhancing crop yield and ecofriendliness	[23]
Nanopesticides	Nanoparticle pesticides enhance effectiveness, reduce required doses, and minimize environmental impact by more precisely targeting pests and reducing harm to nontarget organisms	[24]
Nanosensors	Nanosensors enhance precision agriculture by monitoring soil quality, detecting pests and diseases, and assessing environmental conditions, thereby optimizing resource use and boosting yields	[25]
Nanoencapsulation of bioactive compounds	Encapsulating nutrients, pesticides, or bioactive molecules in nanoscale carriers boosts stability, bioavailability, and targeted delivery, increasing efficiency while minimizing waste and pollution	[26, 27]
Nanoremediation	Nanomaterials clean soil and water by adsorbing, degrading, or immobilizing pollutants, restoring soil fertility and ensuring safe water for agriculture	[28]
Nano-enabled smart delivery systems	Such systems efficiently deliver nutrients, water, or agrochemicals to plants, reducing waste and maximizing resource use	[29]

**Table 2:** Graphics pertinent to agro-nanotechnology in Africa.

**Major graphics**	**Description of the graphics pertinent to agro-nanotechnology in Africa**	**Ref.**
Increased crop yield	Nanofertilizers and nanopesticides enhance crop yields by efficiently delivering nutrients and pest control, outperforming conventional methods, as illustrated by graphics	[34]
Water management	Nanotech sensors monitor soil moisture to optimize irrigation and conserve water, with graphics highlighting their effectiveness	[30]
Soil remediation	Nanomaterials absorb pollutants and enhance soil structure, with graphics illustrating their interaction with contaminants and soil improvement	[35, 36]
Precision agriculture	Nanosensors and nanodevices revolutionize soil monitoring, crop health, and environmental tracking in precision agriculture, with graphics illustrating their integration and benefits for farmers	[37]
Climate smart agriculture	Nanotech enhances climate-smart agriculture by improving resource efficiency and reducing emissions, with graphics showing its role in sustainability and climate resilience	[38, 39]
Food security	Agro-nanotechnology enhances crop productivity and resilience, bolstering food security in Africa, with graphics illustrating its potential to reduce hunger and improve livelihoods through nano-enabled agriculture	[40, 41]
Environmental impact	Visualizations can highlight the environmental benefits of agro-nanotechnology, such as reduced chemical runoff, soil erosion, and pesticide residues, fostering healthier ecosystems	[42, 43]
Economic growth	Graphics can show how agro-nanotechnology adoption boosts economic growth through enhanced agricultural productivity, job creation, and innovation in the sector	[3]

## Data Availability

Data sharing is not applicable to this article, as no new data was analyzed in this study.
